# A novel family of sugar-specific phosphodiesterases that remove zwitterionic modifications of GlcNAc

**DOI:** 10.1016/j.jbc.2023.105437

**Published:** 2023-11-07

**Authors:** Samantha L. Fossa, Brian P. Anton, Daniel W. Kneller, Laudine M.C. Petralia, Mehul B. Ganatra, Madison L. Boisvert, Saulius Vainauskas, Siu-Hong Chan, Cornelis H. Hokke, Jeremy M. Foster, Christopher H. Taron

**Affiliations:** 1 Research Department, New England Biolabs, Ipswich, Massachusetts, USA; 2 Department of Parasitology, Leiden University – Center of Infectious Diseases, Leiden University Medical Center, Leiden, The Netherlands

**Keywords:** phosphorylcholine, phosphodiesterase, functional metagenomics, parasitology, glycobiology, glycan analysis, N-glycans, *N*-acetylglucosamine (GlcNAc), glycosphingolipid

## Abstract

The zwitterions phosphorylcholine (PC) and phosphoethanolamine (PE) are often found esterified to certain sugars in polysaccharides and glycoconjugates in a wide range of biological species. One such modification involves PC attachment to the 6-carbon of *N*-acetylglucosamine (GlcNAc-6-PC) in N-glycans and glycosphingolipids (GSLs) of parasitic nematodes, a modification that helps the parasite evade host immunity. Knowledge of enzymes involved in the synthesis and degradation of PC and PE modifications is limited. More detailed studies on such enzymes would contribute to a better understanding of the function of PC modifications and have potential application in the structural analysis of zwitterion-modified glycans. In this study, we used functional metagenomic screening to identify phosphodiesterases encoded in a human fecal DNA fosmid library that remove PC from GlcNAc-6-PC. A novel bacterial phosphodiesterase was identified and biochemically characterized. This enzyme (termed GlcNAc-PDase) shows remarkable substrate preference for GlcNAc-6-PC and GlcNAc-6-PE, with little or no activity on other zwitterion-modified hexoses. The identified GlcNAc-PDase protein sequence is a member of the large endonuclease/exonuclease/phosphatase superfamily where it defines a distinct subfamily of related sequences of previously unknown function, mostly from *Clostridium* bacteria species. Finally, we demonstrate use of GlcNAc-PDase to confirm the presence of GlcNAc-6-PC in N-glycans and GSLs of the parasitic nematode *Brugia malayi* in a glycoanalytical workflow.

The outer surface of cells is coated with a diverse set of sugar-based polymers called glycans. Glycans are assembled from monosaccharide building blocks into long polysaccharide chains or into smaller but structurally complex oligosaccharides covalently attached to proteins or lipids (glycoconjugates). Adding to this complexity, site-specific enzymatic modifications of glycans with different chemical groups (*e.g*., methyl, sulfate, phosphate, acetyl, or zwitterions) may occur. These modifications are often referred to as “postglycosylational modifications” (PGMs) (1, 2). PGMs act in many contexts as important modulators of glycan biological function. Changes in the PGM content of cellular or tissue glycomes have been observed in many diseases including autoimmune disorders, cystic fibrosis, infections, and certain cancers (2). As such, this area of glycobiology has enormous potential for therapeutic and molecular diagnostic application. However, analysis of PGMs remains technically challenging, in part due to there being few well-characterized and highly selective molecular tools (*e.g*., enzymes, binding proteins) that assist in their detection and simplify their analysis.

One interesting class of PGM consists of zwitterionic modification of glycans. These PGMs involve esterification of phosphorylcholine (PC) or phosphoethanolamine (PE) to a sugar ring (Fig. 1*A*). They are present on a variety of polysaccharides and glycoconjugates throughout biology. In eukaryotes, PC and PE have been observed on N-glycans from several invertebrates (3) and *Penicillium* fungi (4). They are prevalent on N-glycans and glycolipids of nematodes, including several mammalian parasite species (5, 6, 7, 8, 9, 10), where they help the parasite evade host immunity through inhibition of immune cell activity (6). Additionally, the eukaryotic glycosylphosphatidylinositol (GPI) anchor possesses a PE moiety that participates in linking the GPI to protein and further side-branching PE residues decorate its glycan core in mammals and yeasts (11, 12, 13, 14). In bacteria, PE has been found on the cellulosic biofilm of *Escherichia coli* where it likely protects the polymer from degradation by hydrolytic enzymes (15). Additionally, sugar-associated zwitterions have been observed on diacylglycerol in *Clostridium tetani* (16), and in cell wall polysaccharides and teichoic acids of several pathogenic bacteria (17, 18). Despite the breadth of PC/PE distribution in glycobiology, few enzymes that either add (12, 19, 20) or remove (21, 22, 23) these groups from sugars have been defined. Thus, better definition of the range of enzymes involved in manipulating glycan zwitterions will lead to better understanding of the biosynthesis, degradation, and functions of these PGMs and may also have utility in glycoanalytical workflows.

**Figure 1:**
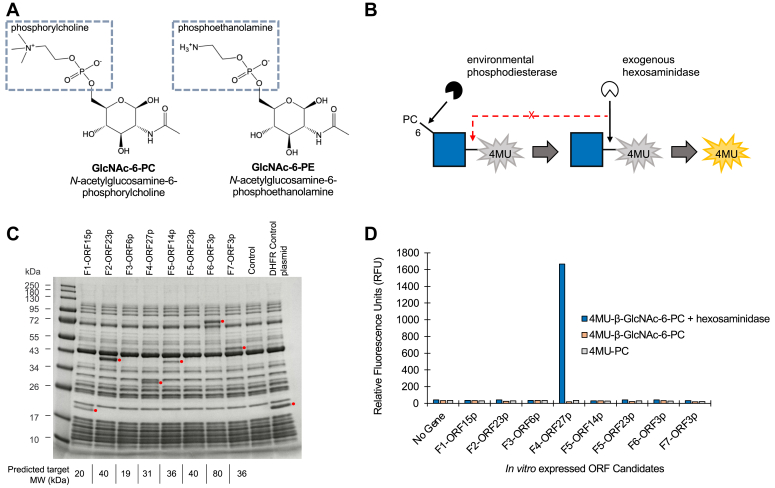
**Metagenomic screening strategy and candidate phosphodiesterase assessment.***A*, chemical structures of GlcNAc-6-PC and GlcNAc-6-PE. *B*, a schematic of the coupled assay used to screen for environmental GlcNAc-6-PC phosphodiesterases. A quenched fluorescent GlcNAc analog (4MU-β-GlcNAc-6-PC) is used as substrate. A fluorescent signal (*yellow* 4-MU) is generated upon sequential removal of PC by an environmental phosphodiesterase and GlcNAc by β-*N*-acetylhexosaminidase_f_ provided in the assay. Screening data is provided in Fig. S1. Glycan structure is represented using the Symbol Nomenclature for Glycans nomenclature. *C*, SDS-PAGE separation of *in vitro* produced proteins from eight candidate ORFs identified in the DNA sequence of fosmid “hits” (see Table 1). Shown are expressed proteins (*red circles*) and the predicted molecular weight (kDa) of each (*bottom*). *D*, proteins were assayed for phosphodiesterase activity using 4MU-β-GlcNAc-6-PC ( ± hexosaminidase) and 4MU-PC substrates. Fluorescence was measured at λ_ex_ = 365 nm and λ_em_ = 445 nm after 3 h. 4MU, 4-methylumbelliferyl; DHFR, dihydrofolate receptor; PC, phosphorylcholine; PE, phosphoethanolamine.

The primary aim of the current study was to use high-throughput functional metagenomic screening to discover enzymes that hydrolyze zwitterions from sugars. Functional metagenomics is a powerful method for detecting enzymatic activities encoded in environmental DNA (eDNA). A typical approach involves eDNA isolation from any environment, cloning large eDNA fragments into fosmid vectors (typically in *E. coli*), and screening clones in high throughput for microexpression of a desired enzyme activity. The main advantages of this method are that enzymes are identified based on their activity (not similarity to known protein families) and that proteins from both cultivable and uncultivable microorganisms can be found. Thus, functional metagenomics is well suited to identify new specificities and often new protein families.

We previously established a functional metagenomic screen to identify enzymes that hydrolyze PGMs, specifically the removal of sulfate from C6 of *N*-acetylglucosamine (GlcNAc) (24). In the current study, we have further adapted this general strategy to seek enzymes that hydrolyze PC from C6 of GlcNAc (GlcNAc-6-PC). This structure is the predominant zwitterion modification of N-glycans and glycolipids in the human parasitic nematode *Brugia malayi* (9) and other filarial parasites. We report successful identification and biochemical characterization of a new family of phosphodiesterases with remarkable selectivity for zwitterion modifications of GlcNAc at C6. We define the biochemical properties and preliminary structural features of multiple members of this protein family. Finally, we show the utility of this enzyme for detection of GlcNAc-6-PC in *B. malayi* N-glycans and glycolipids. Our study establishes the feasibility of using functional metagenomics to identify enzymes that act on zwitterion-modified sugars, defines a new family of sugar-specific phosphodiesterases, and validates an important new enzyme specificity for use in glycoanalytical workflows.

## Results

### A functional metagenomic screen for GlcNAc-6-PC phosphodiesterases

A functional metagenomic screen was designed to find enzymes able to remove PC from C6 of GlcNAc. The assay used for screening was adapted from one we previously used to identify sulfatases that remove sulfate from C6 of GlcNAc (24). In the present version, we used a fluorogenic substrate consisting of GlcNAc with PC esterified to C6 and 4-methylumbelliferone (4-MU) attached to C1 (4MU-β-GlcNAc-6-PC). This assay permits an expressed environmental phosphodiesterase to liberate PC from 4MU-β-GlcNAc-6-PC, after which an exogenous β-hexosaminidase provided in the assay mixture hydrolyzes 4MU-β-GlcNAc to generate a fluorescent signal (Fig. 1*B*). A previously reported library of fosmid clones containing ∼40 kb inserts of human fecal eDNA was used for screening (24).

A total of 6144 fosmid clones were screened resulting in identification of 23 “hits.” Hits were defined as clones yielding an assay signal at least ten SDs above the mean background fluorescence measured for clones carrying only the empty pCC1Fos fosmid vector (Fig. S1). Fosmid DNA was isolated from each hit and subjected to long-read nucleotide sequencing as described in Experimental procedures. The assembled nucleotide sequence of seven fosmid inserts, termed F1-F7, were deposited in GenBank under the accession numbers OQ439824-OQ439830. Each fosmid sequence was analyzed by computational prediction of encoded ORFs using MetaGeneMark and BLASTP analysis of each ORF. In our prior screen for enzymes that remove sulfate from GlcNAc-6-SO_4_, we identified both GlcNAc-specific sulfatases and a hexosaminidase that preferentially hydrolyzes GlcNAc-6-SO_4_ (24). As such, in this study, we focused on nine fosmid ORFs having annotations that suggested possible roles in either manipulation of phosphate or sugar hydrolysis (Table 1). Of these nine ORFs, eight were successfully amplified and subsequently expressed *in vitro* using PURExpress (Fig. 1*C*), and the reactions were assayed on 4MU-β-GlcNAc-6-PC, both with and without exogenous hexosaminidase, and 4MU-PC (Fig. 1*D*). A single protein encoded by ORF27 on fosmid F4 was clearly active on 4MU-β-GlcNAc-6-PC in the presence of exogenous hexosaminidase but showed no activity in its absence or on 4MU-PC (herein referred to as GlcNAc-PDase for this study). In an orthologous analysis, the enzyme was shown by ultra-performance liquid chromatography with inline fluorescence and mass detection (UPLC-FLR-MS) to liberate PC from 4MU-β-GlcNAc-6-PC (Fig. S2), confirming that it was functioning as a phosphodiesterase. Characterization of the biochemical properties of GlcNAc-PDase and its protein family is the subject of the remainder of this study.

**Table 1:** Enzyme candidates identified from functional screening with 4MU-β-GlcNAc-6-PC

Fosmid osmid no.	ORF no.^a^	GenBank ID (fosmid)^b^	GenBank ID (closest neighbor)^c^	NCBI blast annotation (closest neighbor)^d^
Putative phosphate-active enzymes				
F1	ORF15	OQ439824	WP_008663481	alpha-ribazole phosphatase
F2	ORF23	OQ439825	WP_004293565	phosphoesterase
F3	ORF6	OQ439826	WP_227069831	metallophosphatase family protein
F4	ORF27	OQ439827	MBS6702114	endonuclease/exonuclease/phosphatase family protein
F5	ORF14	OQ439828	WP_007051123	Ppx/GppA family phosphatase
Putative glycoside hydrolases				
F5	ORF23	OQ439828	WP_118294042	glycoside hydrolase family 3 protein
F6	ORF3	OQ439829	EEI80631	glycosyl hydrolase family 20 protein
F7	ORF2	OQ439830	MBT9701905	family 43 glycosylhydrolase
F7	ORF3	OQ439830	MBT9701906	family 43 glycosylhydrolase

a An ORF number was generated by MetaGeneMark analysis of each fosmid’s cloned eDNA insert sequence.

b The entire sequence of each fosmid was deposited in GenBank.

c BLASTP analysis of the nonredundant GenBank database was conducted using each deduced ORF peptide sequence as a query. Shown is the GenBank number and annotation of the most highly related protein from each search.

d BLASTP analysis of the nonredundant GenBank database was conducted using each deduced ORF peptide sequence as a query. Shown is the GenBank number and annotation of the most highly related protein from each search.

### Analysis of the GlcNAc-PDase protein sequence and sequence family

Basic features of the deduced GlcNAc-PDase sequence were analyzed computationally. The transmembrane prediction algorithm TMHMM 2.0 and the signal peptide prediction tool SignalP 5.0 revealed that GlcNAc-PDase lacks transmembrane segments and possesses a signal peptide (predicted cleavage after glycine-34: VPG↓SG), suggesting it is likely a soluble secreted protein in its native environment. Protein BLAST against the GenBank nonredundant database revealed that the two most similar proteins were both from Clostridiales bacteria (accession numbers MBS6702114 and MBS6250406, with 99% and 94% identity, respectively). These uncharacterized proteins are members of a very large endonuclease/exonuclease/phosphatase (EEP) superfamily (Pfam protein family PF03372 and Conserved Domain Database family cd08372). Characterized proteins in this diverse enzyme superfamily share a common catalytic mechanism that breaks phosphodiester bonds. Members include enzymes that act upon nucleic acids such as certain apurinic/apyrimidinic endonucleases and DNase I exonucleases and enzymes that act upon certain phosphodiester-linked lipids like sphingomyelin.

**Figure 2:**
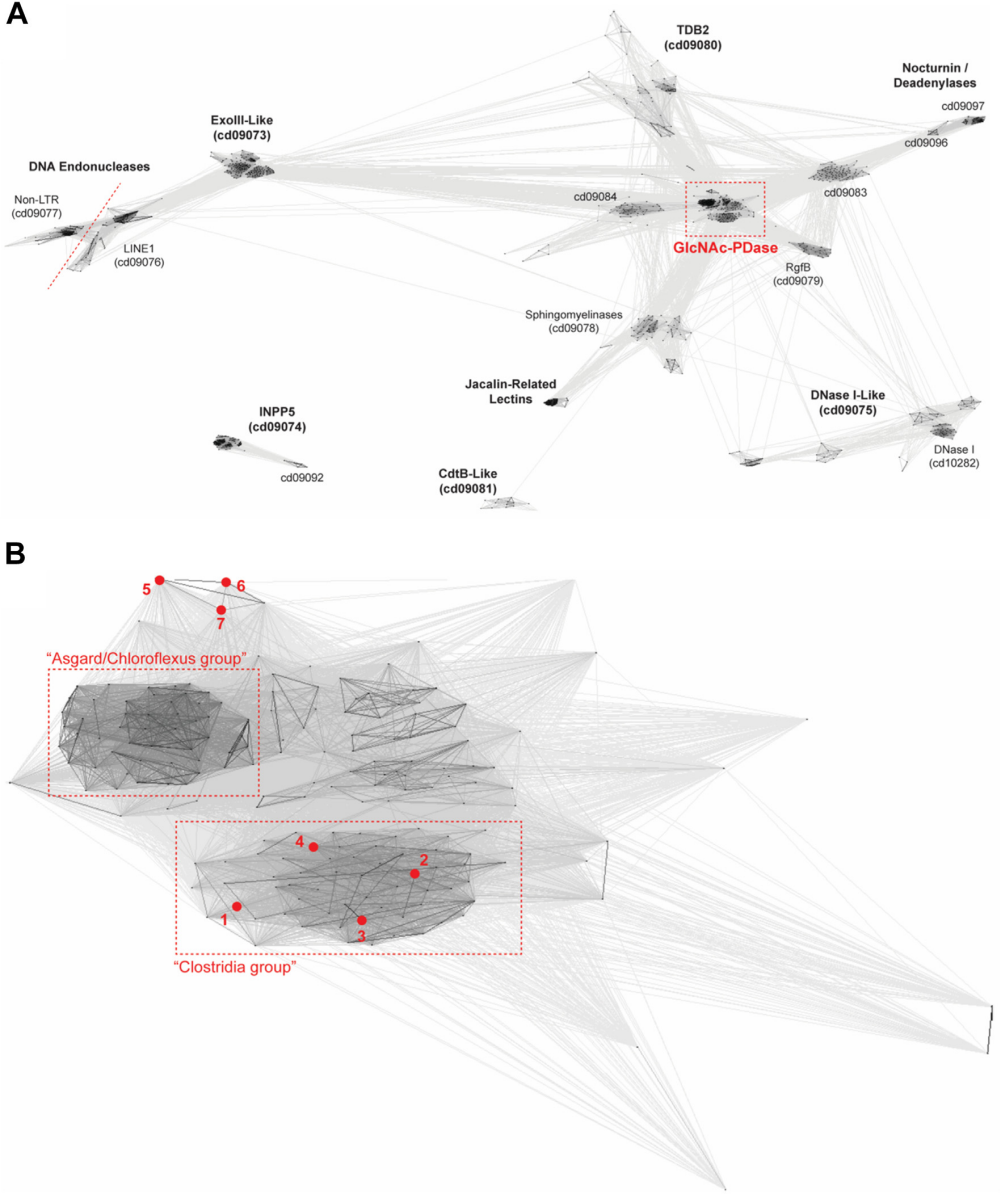
**GlcNAc-PDase sequence clustering analysis within EEP superfamily.** Results of sequence clustering of EEP protein sequences using CLANS (38). Each protein is represented by a node (point). Edges are drawn between nodes with sufficient pairwise sequence similarity, as determined by BLAST *p*-value; in general, the darker the edge, the greater the similarity. Groups of proteins with a mutually high degree of relatedness will tend to cluster. *A*, clustering of 900 proteins from throughout the EEP superfamily. Clusters are labeled with the family name based on characterized and/or named members and, where defined, the CDD family number. Full-length protein sequences are used, and so for multidomain proteins, some edges may be formed or strengthened based on similarity outside the EEP domain. The *red box* indicates the GlcNAc-PDase group, a unique and defined cluster that does not have an assigned CDD number. *B*, clustering of 186 proteins from the GlcNAc-PDase group, now trimmed to restrict the sequences to the EEP domain only. Two taxonomically specific clusters are indicated by the *red boxes*, one comprising sequences primarily from Asgardarchaeota (archaea) and Chloroflexota (bacteria) and the other comprising sequences primarily from Clostridia (bacteria). Seven nodes (proteins) are labeled by number, including the four proteins experimentally characterized in this work [#1–4] and three previously studied eukaryotic proteins [#5–7]: [1] GlcNAc-PDase from an unknown strain, [2] HCG67795 from Clostridiales bacterium, [3] MBE6669927 from Ruminococcaceae bacterium, [4] NLD61619 from Candidatus Sumerlaeota bacterium, [5] Cwh43p from *Saccharomyces cerevisiae*, [6] Cwh43p from *Schizosaccharomyces pombe*, and [7] PGAP2IP from *Mus musculus*. CDD, Conserved Domain Database; EEP, endonuclease/exonuclease/phosphatase.

A sequence clustering analysis was performed to determine the relatedness of GlcNAc-PDase to 900 other sequences from the EEP superfamily. GlcNAc-PDase clusters closely with groupings of protein sequences (cd09079, cd09083, cd09084) of unknown function and sphingomyelinases (cd09078) (Fig. 2*A*). Within the immediate GlcNAc-PDase cluster, only two proteins, yeast Cwh43p and human PGAP2IP, have been previously studied (25, 26). These two related proteins have been implicated in lipid remodeling events during biosynthesis of GPI anchors, however, the precise role of their EEP domains is still vague. Thus, sequence cluster analysis points to the GlcNAc-PDase group as being a unique and distinct subfamily of PF03372.

The GlcNAc-PDase group was further stratified by clustering its 187 sequences (Fig. 2*B*). Two prominent, taxonomically restricted subclusters, one including mostly proteins from Asgardarchaeota (archaea) and Chloroflexota (bacteria) and the other including mostly proteins from Clostridia (bacteria) are noted. However, the functional significance, if any, of the subclusters within the GlcNAc-PDase group is unclear. The “Clostridia group,” including those with the highest similarity to GlcNAc-PDase, comprises 65 sequences. Most member proteins are 219 to 357-aa long and are composed nearly entirely of a single EEP domain with a secretion peptide and no transmembrane segments. While not biochemically explored in this study, proteins in the “Asgard/Chloroflexus group” were divergent from the Clostridia group in their size and domain organization.

### Biochemical properties of GlcNAc-PDase

**Figure 3:**
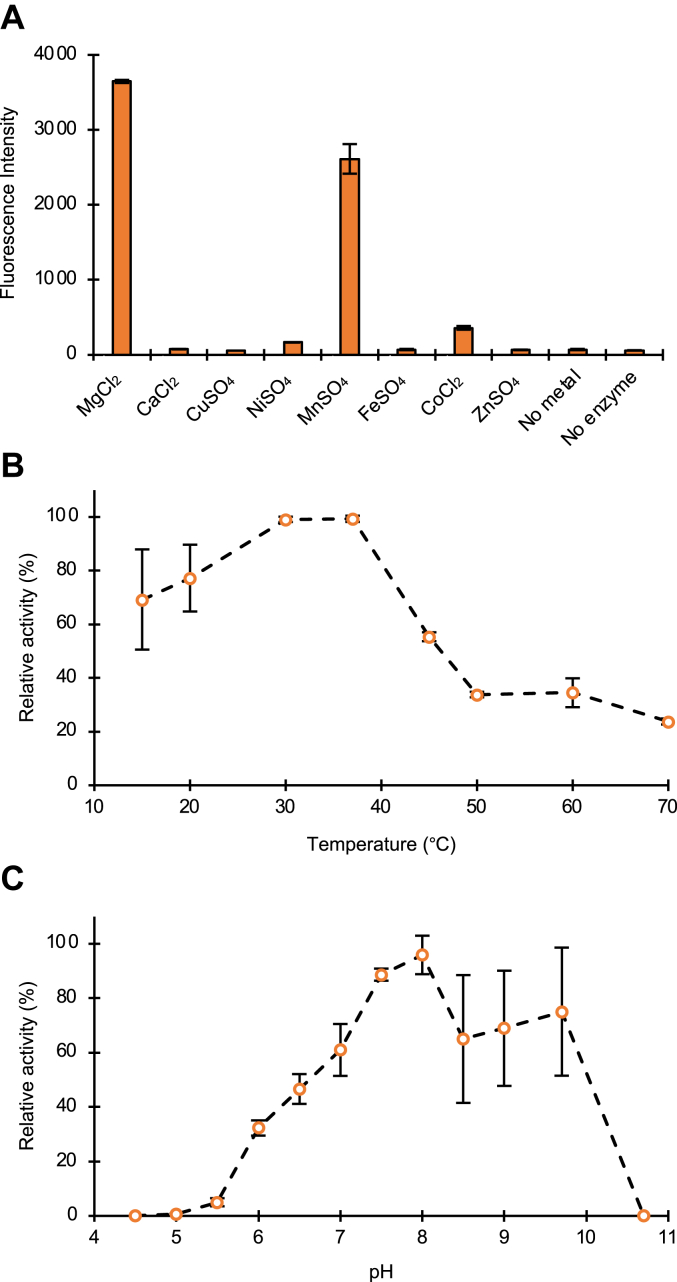
**GlcNAc-PDase-8His biochemical characterization.***In vivo* produced and purified GlcNAc-PDase-8His was used to define the enzyme’s catalytic properties. *A*, the substrate 4MU-β-GlcNAc-6-PC was used to determine GlcNAc-PDase activity in the presence of metal ions. Procainamide-labeled GlcNAc-6-PC was used as substrate to determine GlcNAc-PDase-8His optimal (*B*) temperature and (*C*) pH. All reactions were performed in triplicate. 4MU, 4-methylumbelliferyl; PC, phosphorylcholine.

To define the basic biochemical properties of GlcNAc-PDase, its recombinant production and purification was undertaken. His-tagged GlcNAc-PDase (GlcNAc-PDase-8His) was produced in *E. coli* and purified using nickel affinity chromatography as described in Experimental procedures (Fig. S3). Purified GlcNAc-PDase-8His required a divalent metal ion for catalysis *in vitro* (Fig. 3*A*). It was most active in the presence of Mg^2+^ and Mn^2+^ ions, and to a lesser degree, Ni^2+^ and Co^2+^ ions. The enzyme was active over a broad temperature range (15–60 °C) with an optimum from 30 to 37 °C (Fig. 3*B*) and was active from pH 6 to 10 (Fig. 3*C*). Applying these biochemical properties, a unit was defined as the amount of enzyme needed to liberate PC from 10 nmol of GlcNAc-6-PC in 50 mM Tris–HCl, pH 8 containing 5 mM MgCl_2_ in 1 h at 37 °C.

### Specificity of selected “Clostridia group” proteins

The specificity of GlcNAc-PDase and other selected proteins from the Clostridia group was explored *in vitro*. For this experiment, three proteins from the Clostridia group were selected for functional comparison to GlcNAc-PDase. These proteins are referred to by their GenBank accession numbers as follows: NLD61619, from Candidatus Sumerlaeota bacterium; MBE6669927, from Oscillospiraceae bacterium; and HCG67795, from Clostridiales bacterium. These proteins consist of 262 to 297 aa, have a single EEP domain, and share 34%, 38%, and 38% identity with GlcNAc-PDase, respectively (See Fig. S4 for additional information). Recombinant production of each was achieved by *in vitro* transcription and translation (IVTT) using the PURExpress system (Fig. S5*A*). IVTT-produced material was assayed for activity on the screening substrate 4MU-β-GlcNAc-6-PC with β-hexosaminidase to confirm that it was functional (Fig. S5*B*).

**Figure 4:**
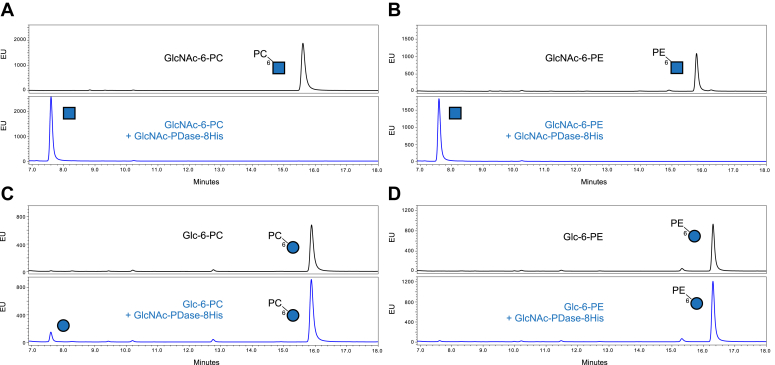
**GlcNAc-PDase-8His specificity analyzed by UPLC-FLR.***In vivo* produced and purified GlcNAc-PDase-8His was incubated with (*A*) GlcNAc-6-PC, (*B*) GlcNAc-6-PE, (*C*) Glc-6-PC, or (*D*) Glc-6-PE for 20 h, procainamide-labeled, and then separated by UPLC-FLR. For each set of traces, the *top panel* shows a control reaction with substrate alone and the *bottom panel* shows digestion of the substrate by GlcNAc-PDase-8His. Glycan structures are represented using the Symbol Nomenclature for Glycans nomenclature: *blue circle*, glucose; *blue square*, GlcNAc; Numbers reflect the sugar carbon that is bonded with the zwitterion. FLR, fluorescence; PC, phosphorylcholine; PE, phosphoethanolamine; UPLC, ultra-performance liquid chromatography.

The specificity of the four enzymes was explored using a panel of synthetic monosaccharides possessing zwitterions. In these assays, IVTT-produced enzyme was incubated with a monosaccharide substrate, after which, the sugar was reductively labeled with procainamide. The reaction products were then analyzed by UPLC-FLR. The ability of each enzyme to remove PC or PE from eight different substrates was determined (Table 2; Figs. S6–S13). The four IVTT-produced enzymes performed similarly with a marked preference for removal of PC or PE from the 6-carbon of GlcNAc (Figs. S6 and S10). This preference was also observed with purified GlcNAc-PDase-8His that was produced *in vivo* (Fig. 4, *A* and *B*). Significantly lower hydrolysis of Glc-6-PC was observed for all four proteins, and only related proteins 1 (NLD61619) and 2 (MBE6669927) showed lower hydrolysis of Glc-6-PE (Figs. 4, *C* and *D*, S7, and S11). All four enzymes were each unable to efficiently remove PC or PE from the 6-carbon of galactose or mannose, or PE from the 2-carbon of mannose (a linkage found in fungal and mammalian GPI anchors) (Figs. S8, S9, S12, and S13). These data support the conclusion that the Clostridia group cluster of proteins represents a family of highly specific GlcNAc-preferring phosphodiesterases.

**Table 2:** Activity of “Clostridia group” enzymes on synthetic zwitterionic monosaccharide substrates.

Monosaccharide Substrate^1^	Substrate Structure^2^	*In vitro* Produced Clostridia Group Proteins^3^			
		GlcNAc-PDase	Related protein 1 (NLD61619)	Related protein 2 (MBE6669927)	Related protein 3 (HCG67795)
GlcNAc-6-PC		+++^4^	+++	+++	+++
Glc-6-PC		+	++	++	+
Gal-6-PC		-	-	-	-
Man-6-PC		-	-	-	-
GlcNAc-6-PE		+++	+++	+++	+++
Glc-6-PE		-	+	++	-
Man-6-PE		-	-	-	-
Man-2-PE		-	-	-	-

1 Monosaccharide abbreviations: Glc, glucose; Gal, galactose, and Man, mannose. Zwitterion abbreviations: PC, phosphorylcholine and PE, phosphoethanolamine. Numbers reflect the sugar carbon that is bonded with the zwitterion.

2 Glycan structures are represented using the Symbol Nomenclature for Glycans nomenclature: blue circle = Glc, blue square = GlcNAc, yellow circle = Gal, green circle = Man.

3 Proteins other than GlcNAc-PDase are referred to by their GenBank accession number. The data in this table was collected with enzymes produced using IVTT. Because the concentration of IVTT-expressed enzymes cannot easily be standardized to each other, the amount of active enzyme assayed may vary between the proteins. Thus, while these data accurately reflect the ability of each protein to hydrolyze each substrate, it should not be used to quantitatively compare efficiencies between the enzymes for any given substrate.

4 After incubation of each substrate with each enzyme, monosaccharides were labeled with procainamide and analyzed by UPLC-FLR. The amount of product generated was quantified by integration of the observed peaks; “-”, 0 to 10% digestion; “+”, 11 to 40% digestion; “++”, 41 to 90% digestion; and “+++”, 91 to 100% digestion. All UPLC-FLR traces are provided in Supporting information Figures S6-S13.

### Structural modeling of GlcNAc-PDase

**Figure 5:**
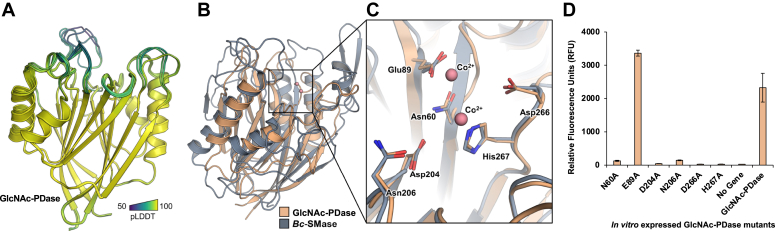
**Structural analysis of GlcNAc-PDase.***A*, predicted structure of the GlcNAc-PDase sequence in cartoon representation colored in Viridis by predicted Local Distance Difference Test (pLDDT) scores. The structured regions from residues 50 to 278 for ranks 1 to 5 are shown. For rank 1, per-residue estimate confidence scores for the structured regions from residues 50 to 278 have a mean ± SD of 96 ± 4.4. *B*, superposition of the GlcNAc-PDase predicted structure (*light orange*) with a 1.8 Å X-ray crystal structure of *Bacillus cereus* sphingomyelin phosphodiesterase (*Bc*-SMase) in complex with two cobalt ions (*blue-gray*, Co^2+^ as *salmon spheres*). *C*, metal ion–binding site of GlcNAc-PDase and *Bc*-SMase. Residues are shown as *sticks* with the GlcNAc-PDase sequence used for position numbers. *D*, activity of GlcNAc-PDase metal-binding site alanine mutants. Six mutants of GlcNAc-PDase (N60A, E89A, D04A, N206A, D266A, and H267A) were produced *in vitro*. For each mutant, fluorescence release from 4MU-β-GlcNAc-6-PC in the presence of exogenous hexosaminidase was assessed after 1 h in triplicate reactions. Mutants were compared to a no gene control and WT GlcNAc-PDase. PC, phosphorylcholine.

The tertiary structure of GlcNAc-PDase was predicted with AlphaFold2 to provide insight into the catalytic structure-function relationship (Fig. 5*A*). The top five models displayed consistent and confident scores in the predicted Local Distance Difference Test (pLDDT) (27), suggesting reliable backbone predictions for most of the protein and permitting investigation of active site residues.

The predicted coordinates for GlcNAc-PDase were first queried against experimental structures to identify homologous folds using the DALI protein structure comparison server (28, 29). Over 50 X-ray structures in the PDB90 representative database returned high Z-scores (>10) and low-sequence identity (<25%). An X-ray crystal structure of Sphingomyelin phosphodiesterase from *Bacillus cereus* (*Bc*-SMase, PDB 2DDS) was used for detailed structural comparison with the GlcNAc-PDase model (30).

Both proteins possess an overall β-sandwich–like fold comprised of β-α-β motifs. The GlcNAc-PDase structural region aligns to *Bc*-SMase with a Cα RMSD of ∼4 Å indicating global structural homology (Fig. 5*B*). As EEP superfamily phosphodiesterase enzymes, both GlcNAc-PDase and *Bc*-SMase activity are dependent on divalent metal ion binding. In the *Bc*-SMase crystal structure, two Co^2+^ ions are coordinated by six residues in a conserved cleft that has been proposed to directly position the substrate scissile phosphodiester bond for hydrolytic cleavage (30). The *Bc*-SMase metal-binding residues are in near-identical positions with homologous residues of GlcNAc-PDase implying the latter also coordinates two metal ions for correct scissile bond orientation (Fig. 5*C*). Indeed, single site-directed substitutions to alanine for these residues significantly diminish the phosphodiesterase activity of GlcNAc-PDase on 4MU-β-GlcNAc-6-PC, except for Glu89 (Fig. 5*D*). Predicted structures of the three tested homologous enzymes selected from the Clostridia group sequence cluster (Table 2) also possess structural conservation for this metal ion–binding site (Fig. S14).

### Detecting GlcNAc-6-PC in parasitic nematode glycans

The ability of GlcNAc-PDase to remove PC from GlcNAc-6-PC from intact glycans was investigated. Recent characterization of the glycome of the filarial parasitic nematode *B*. *malayi* revealed the presence of glycans bearing zwitterionic sugar modifications on both GlcNAc and mannose (Man-PC) (9). In this study, GlcNAc-6-PC was observed in the terminal position of N-glycan outer arms and in glycosphingolipids (GSLs). Thus, we explored if GlcNAc-PDase could remove PC from GlcNAc-6-PC in intact *B. malayi* glycans. Four fractions containing glycans obtained from *B. malayi* were used as substrates for digestion with IVTT-produced GlcNAc-PDase. Reaction products were analyzed by MALDI-TOF mass spectrometry (MS).

**Figure 6:**
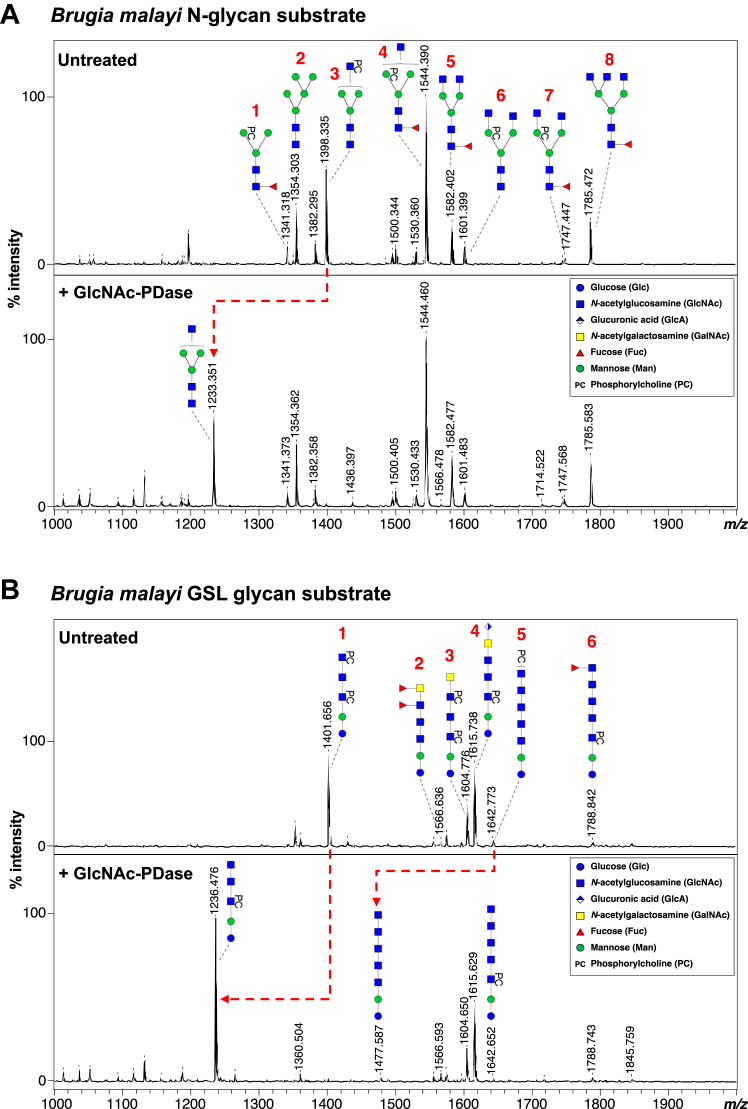
**Digestion of *Brugia malayi* glycans with GlcNAc-PDase on MALDI-TOF-MS.** Enzymatically released, AA-labeled and UHPLC purified *B. malayi* (*A*) N-linked and (*B*) GSL glycans were subjected to digestion with GlcNAc-PDase. Resulting digestion products are highlighted using *red dashed arrows*. MALDI-TOF-MS spectra monoisotopic masses are indicated for glycan ions and glycan structures are represented using the CFG nomenclature. See Symbol key inset. MALDI-TOF-MS raw data can be found in Table S2*A*. AA, aminobenzoic acid; GSL, glycosphingolipid; MS, mass spectrometry; UHPLC, ultrahigh performance liquid chromatography.

Our *B. malayi* N-glycan substrate has a MALDI ion profile consisting of eight previously characterized N-glycans species (Fig. 6*A*). Of these, four N-glycans contain PC-modified mannose (structures 1, 4, 6, and 7) and one has a terminal GlcNAc-6-PC (structure 3). Following GlcNAc-PDase digestion, only the ion signature of structure 3 changed (from *m*/*z* 1398.335 [M-H]^-^ to *m*/*z* 1233.351 [M-H]^-^) consistent with loss of a single PC (Δ165 Da) from its terminal GlcNAc. No *m*/*z* changes were observed for any of the Man-PC structures. Thus, GlcNAc-PDase was able to access and remove PC from intact N-glycans and shows the same GlcNAc selectivity observed in our monosaccharide experiments (above).

The first *B. malayi* GSL glycan substrate contains six different structures, five of which harbor GlcNAc-6-PC (Fig. 6*B*; structures 1, 3, 4, 5, and 6). Upon digestion with GlcNAc-PDase, the *m*/*z* values of structures 1 and 5 each shifted by 165 Da due to the loss of PC. Interestingly, the signatures of GlcNAc-6-PC–containing structures 3, 4, and 6 were unaltered. These structures each have PC esterified to a GlcNAc that is internal to the glycan, suggesting that GlcNAc-6-PC must be in a terminal position for PC to be removed by GlcNAc-PDase. It is also noteworthy that only partial digestion of structure 5 was observed, suggesting this glycan fraction comprises a mixture of isomeric structures for which not all GlcNAc-6-PC is in the terminal position.

We further assessed the ability of GlcNAc-PDase to be used in conjunction with an exoglycosidase to verify the presence of internal GlcNAc-6-PC in a GSL glycan. In this experiment, a second GSL glycan substrate having three GlcNAc-6-PC–containing structures was used (Fig. S15*A*). Treatment with β-*N*-acetylhexosaminidase_f_ resulted in the removal of unsubstituted GalNAc and GlcNAc residues from structures 1 and 3, respectively, to yield structure 4 (*m*/*z* 830.276 [M-H]^-^) that possesses terminal GlcNAc-6-PC (Fig. S15*B*.i). Structure 4 became further susceptible to digestion with hexosaminidase only after treatment with GlcNAc-PDase (Fig. S15*B*.ii). These data again support the idea that GlcNAc-6-PC must be in a terminal position for PC to be removed by GlcNAc-PDase. A similar experiment was conducted with an N-glycan substrate that bears terminal Man-PC (*m*/*z* 1033.365 [M-H]^-^) (Fig. S16*B*.i). No digestion of this glycan was observed after sequential treatment with α1−2,3,6 mannosidase and GlcNAc-PDase (Fig. S16*B*.ii), confirming that GlcNAc-PDase does not act upon Man-PC, either in a terminal or internal position. Together, these data illustrate the analytical application of GlcNAc-PDase with and without exoglycosidases to verify the presence and location of GlcNAc-6-PC within a glycan.

## Discussion

In the present study, we used functional metagenomic screening to identify active phosphodiesterases produced by human gut microbes that remove zwitterions from carbon-6 of GlcNAc. We describe discovery of a new family of sugar-specific phosphodiesterases having a unique specificity and that functionally define a previously uncharacterized sequence cluster within the large and diverse EEP protein superfamily. We characterized the specificity and biochemical requirements of several members of this protein subfamily and demonstrate an analytical application of this specificity to confirm the presence of GlcNAc-6-PC on glycans, isolated from the parasitic nematode *B*. *malayi*.

Despite the wide distribution of zwitterionic modifications of glycans and glycolipids, few phosphodiesterases that specifically remove these groups from sugars have been described. In yeast and mammalian GPI anchoring, proteins have been implicated in hydrolysis of side-branching PE residues from the GPI glycan. A recombinant version of the mammalian protein PGAP5 can remove PE from the second GPI mannose (Man-2) *in vitro* (21). Yeast proteins with similarity to PGAP5 (Ted1p and Cdc1p) have also been genetically implicated in removal of PE from the innermost two mannoses of the GPI glycan (22, 31). All three proteins share some sequence similarity (22) and each are members of the MMPE1 metallophosphatase family (cd081165). In a second example, a PC esterase from *Streptococcus pneumoniae* removes PC from GalNAc in pneumococcal teichoic and lipoteichoic acid (23). PC esterase is a member of the MBL-fold metallohydrolase (cd07731) family. None of the aforementioned proteins share sequence similarity with GlcNAc-PDase, and none are members of the EEP superfamily.

An interesting biochemical property of GlcNAc-PDase and its close homologs is their high degree of sugar selectivity. Assays performed using several synthetic zwitterion-modified monosaccharide substrates showed that GlcNAc-PDase has a marked preference for GlcNAc-6-PC or GlcNAc-6-PE substrates with significantly weaker activity on Glc-6-PC or Glc-6-PE. This suggests that 2-aminoacetylation of the glucose ring significantly benefits efficient catalysis. Additionally, mannose (a C2 epimer of glucose) and galactose (a C4 epimer of glucose) bearing zwitterions at C6 were not recognized as substrates further suggesting that GlcNAc-PDase likely discriminates its substrate through interaction with hydroxyls at C2 and C4 of GlcNAc. The notion of sugar selectivity is further supported by the inability of GlcNAc-PDase to hydrolyze the sugarless substrate 4MU-PC, indicating that the presence of a PC phosphodiester linkage alone is not sufficient to permit catalysis. Together, these observations support the conclusion that GlcNAc-PDase and the related proteins in the Clostridia group are sugar-specific phosphodiesterases with remarkable selectivity for GlcNAc-6-PC or GlcNAc-6-PE.

Recent advances in protein structure prediction allowed for comparative analysis of high-confidence models of GlcNAc-PDase with the *Bc*-SMase-Co^2+^ complex crystal structure (32, 33). GlcNAc-PDase and selected homologs possess structurally conserved EEP metal-binding residues with *Bc*-SMase that likely coordinate divalent metal ions in the active site for hydrolytic activity (30) as supported by mutagenesis. However, determining the structural basis for zwitterion recognition and the sugar selectivity displayed by this enzyme family will require experimental structure determination in complex with metal ions and substrate molecules.

Analysis of glycans containing zwitterions has historically been challenging. Depending on a variety of technical factors (*e.g*., ionization mode, instrument resolution), MS alone can easily miss the presence of a zwitterion on a glycan or fail to discriminate it from other modifications like sulfate (nicely discussed in reference 3). Evidence of PC or PE presence on a glycan can be bolstered by chemical removal of phosphate esters using hydrofluoric acid treatment. However, this can also remove other sugars like fucose and galactofuranose and is not applicable to experiments involving living cells. Thus, sugar-specific phosphodiesterases, used in conjunction with additional exoglycosidases, represent compelling tools for glycan sequencing workflows to confirm the presence of zwitterions. The demonstrated ability of GlcNAc-PDase to hydrolyze PC from GlcNAc-6-PC of intact N-glycans and GSLs suggests its potential for *in situ* analysis of parasite cell surfaces, where GlcNAc is a prevalent modification, and better definition of glycans at the host-pathogen interface.

## Experimental procedures

Chemicals, reagents, and enzymes β-*N*-acetylhexosaminidase_f_, RNase inhibitor murine, Q5 Hot Start High-Fidelity 2× Master Mix, endoglycoceramidase I (EGCase I), NEB Express *I*^*q*^ Competent *E. coli, N*-Glycosidase F (PNGase F) (glycerol-free, recombinant), α1-2,3,6 mannosidase, and GlycoBuffer 1 were from New England Biolabs. 4-Methylumbelliferyl *N*-acetyl-β-D-glucosaminide-6-phosphorylcholine (4MU-β-GlcNAc-6-PC) and 4-Methylumbelliferyl phosphorylcholine (4MU-PC) were from Biosynth. 4MU-β-GlcNAc was from Dextra Laboratories Ltd. *N*-acetyl-D-glucosamine-6-phosphorylcholine (GlcNAc-6-PC) and *N*-acetyl-D-glucosamine-6-phosphoethanolamine (GlcNAc-6-PE) were synthesized by Creative Biolabs. 6-O-phosphorylcholine-D-glucopyranose (Glc-6-PC), 6-O-phosphorylcholine-D-galactopyranose (Gal-6-PC), 6-O-phosphorylcholine-D-mannopyranose (Man-6-PC), 6-O-phosphoethanolamine-D-glucopyranose (Glc-6-PE), 6-O-phosphoethanolamine-D-mannopyranose (Man-6-PE), and 2-O-phosphoethanolamine-D-mannopyranose (Man-2-PE) were synthesized by GlycoUniverse. Y-PER Yeast Protein Extraction Reagent was from Thermo Fisher Scientific Inc. The CopyControl Fosmid autoinduction solution was from Lucigen Corporation. The Bruker Peptide Calibration Standard and 2,5-dihydroxybenzoic acid (DHB) was from Bruker Daltonics.

### Construction of a human gut microbiome metagenomic library

A fosmid metagenomic library was constructed using DNA extracted from a human gut microbiome fecal sample as previously described (24). Briefly, DNA was isolated from 100 mg of fecal sample. Isolated DNA was used to construct a fosmid library using the CopyControl Fosmid Library Production kit (Lucigen Corporation) following the manufacturer’s instructions.

### Phosphodiesterase screening from a human gut microbiome metagenomic library

A total of 6144 fosmid clones from a human gut microbiome library (24) were screened for GlcNAc-6-PC phosphodiesterase activity in a 384-well plate format. Library clones were cultured overnight at 37 °C in 50 μl Luria–Bertani containing 12.5 μg/ml chloramphenicol and 1× CopyControl Fosmid autoinduction solution. Cultures were lysed by addition of 50 μl of Y-PER Reagent supplemented with 40 μg/ml of 4MU-β-GlcNAc-6-PC and 1 U/ml of β-*N*-acetylhexosaminidase_f_ per well. Reactions were incubated at 37 °C for 48 h. Fluorescence was read at λ_ex_ = 365 nm and λ_em_ = 445 nm using a SpectraMax Plus 384 Microplate reader (Molecular Devices) at different timepoints (1, 5, 24, 48 h). Hits were defined as relative fluorescence units values above three SDs from the mean of the background fluorescence. Hits from the primary screen were validated by repeating the screening protocol. The hit definition was then refined to wells with relative fluorescence units values that were ten SDs above the mean in at least two timepoints.

### Fosmid sequencing

Fosmids from each hit were isolated by BioS&T or using the FosmidMAX DNA Purification Kit (Lucigen Corporation). Isolated fosmids were prepared for Illumina sequencing using the plexWell 96 kit (seqWell) following the manufacturer’s instructions. Sequence assembly was performed by seqWell. ORFs were predicted using MetaGeneMark (34).

### *In vitro* expression of enzyme candidates and mutants

Interesting ORF candidates were selected for *in vitro* expression based on their predicted annotation from a BLASTP search. Selected candidate proteins were produced *in vitro* using the PURExpress *In vitro* Protein Synthesis Kit (New England Biolabs) following the manufacturer’s instructions. Briefly, DNA templates were prepared by PCR using Q5 Hot Start High-Fidelity 2× Master Mix in a 60 μl total reaction volume. Primers used are in Table S1. PCR products were purified using the Monarch PCR & DNA clean-up kit (New England Biolabs). *In vitro* expression was performed for 2 h at 37 °C. A positive control PURExpress reaction was performed in 25 μl total reaction volume using 2 μl of 125 ng/μl dihydrofolate receptor control plasmid supplied with the kit. Expressed proteins were separated on a Novex 10 to 20% Tris-glycine gel (Thermo Fisher Scientific Inc) using 2.5 μl of the reaction. PURExpress reactions were then assayed for phosphodiesterase activity by mixing 7 μl of PURExpress mixtures with 2 μl of 100 μg/ml 4MU-β-GlcNAc-6-PC, and 5 U of β-*N*-acetylhexosaminidase_f_. To assess hexosaminidase activity expressed from fosmid clones, reactions were assayed as indicated above, but in the absence of exogenous β-*N*-acetylhexosaminidase_f_. Reactions were incubated for 1 h at 37 °C and fluorescence was read at λ_ex_ = 365 nm and λ_em_ = 445 nm using a SpectraMax Plus 384 microplate reader.

Six GlcNAc-PDase mutants (N60A, E89A, D204A, N206A, D266A, and H267A) were synthesized with *E. coli* codon optimization and cloned into pUC57 (GenScript). Mutant proteins were produced *in vitro* as described above. Activity of each mutant was assayed on 4MU-β-GlcNAc-6-PC in triplicate as described above for WT GlcNAc-PDase.

### *In vivo* expression and purification of recombinant GlcNAc-PDase-8His

DNA encoding full-length GlcNAc-PDase with a C-terminal 8-His tag was codon optimized for *E. coli* and synthesized (GenScript). This construct was subcloned into expression vector pJS119k (35). The signal peptide was then removed from this construct (aa 2–34) using site-directed mutagenesis (Q5 Site-Directed Mutagenesis Kit, New England Biolabs) to yield pJS119k-GlcNAc-PDase-8His. Luria–Bertani medium supplemented with 25 μg/ml kanamycin was inoculated with NEB Express *I*^*q*^ Competent *E. coli* cells carrying the pJS119k-GlcNAc-PDase-8His plasmid and grown at 37 °C until the A_6__00_ reached 0.4. IPTG was then added to induce expression and the culture was incubated at 16 °C overnight with shaking. Cells were harvested by centrifugation, resuspended in column buffer A (50 mM Tris–HCl, pH 7.5, 500 mM NaCl, and 30 mM imidazole) and lysed by passing twice through a Shear Jet HL60 homogenizer (Dihydromatics) at 25 KPsi. The lysate was loaded onto a HisTrap FF column (Cytiva Life Sciences) that had been pre-equilibrated with column buffer A. The column was washed with column buffer A and bound protein was eluted with column buffer B (50 mM Tris–HCl, pH 7.5, 500 mM NaCl, and 0.5 M imidazole). Fractions containing purified recombinant GlcNAc-PDase-8His were pooled and dialyzed into dialysis buffer (20 mM Tris–HCL, pH 8, 300 mM NaCl, 1 mM EDTA, and 1 mM tris(2-carboxyethyl)phosphine).

### GlcNAc-PDase-8His biochemical characterization

To assess the metal ion requirement of the enzyme, 0.8 U of *in vivo* produced GlcNAc-PDase-8His was incubated with 1 nmol of 4MU-β-GlcNAc-6-PC, 5 U of β-*N*-acetylhexosaminidase_f_ in 50 mM Tris, pH 7.5, with 5 mM of MgCl_2_, CaCl_2_, CuSO_4,_ NiSO_4_, MnSO_4_, FeSO_4_, CoCl_2_, or ZnSO_4_ for 1 h at 37 °C. Reactions were performed in triplicate. Fluorescence was read at λ_ex_ = 365 nm and λ_em_ = 445 nm using a SpectraMax Plus 384 microplate reader.

To determine the optimal pH, 0.8 U of GlcNAc-PDase-8His was incubated for 1 h at 37 °C with 10 nmol GlcNAc-6-PC, 5 mM MgCl_2_, and buffers ranging from pH 4.5 to 10.7 (from pH 4.5–5.5, 50 mM sodium acetate; from pH 6.0–7.0, 20 mM sodium phosphate; from pH 7.5–9, 50 mM Tris–HCl; pH 9.7, 20 mM N-cyclohexyl-3-aminopropanesulfonic acid; and pH 10.7, 50 mM carbonate buffer) in a 10 μl final reaction volume. Reactions were performed in triplicate. After 1 h incubation at 37 °C, 5 μl of each reaction was mixed with 20 μl of 100% acetonitrile (ACN) and dried using a centrifuge concentrator (Vacufuge plus, Eppendorf) for 30 min. Each dried reaction was procainamide-labeled. Procainamide solution was prepared by first dissolving 12 mg of procainamide in 110 μl of 70% dimethyl sulfoxide/30% acetic acid solution. This mixture and 25 μl of water were then transferred to a separate tube containing 6 mg of sodium cyanoborohydride. Dried GlcNAc-PDase reactions were incubated for 1 h at 65 °C with 20 μl freshly made procainamide solution. Reactions were diluted 10-fold in water. A 1 μl aliquot was diluted in 9 μl of 100% ACN and separated by UPLC (see below).

The optimal temperature of GlcNAc-PDase-8His catalysis was determined by incubating 0.8 U GlcNAc-PDase-8His with 10 nmol GlcNAc-6-PC, 5 mM MgCl2, and 50 mM Tris–HCl pH 8.0 for 1 h at 8 temperatures ranging from 15 to 70 °C. Reactions were performed in triplicate, dried, procainamide-labeled, and diluted 10-fold as previously described. A 1 μl aliquot was diluted in 9 μl of 100% ACN and separated by UPLC (see below).

### Ultra-performance liquid chromatography and mass spectrometry

Procainamide-labeled samples were separated by UPLC using a ACQUITY UPLC glycan BEH amide column 130 Å (2.1 × 150 mm, 1.7 μm) from Waters on a H-Class ACQUITY instrument (Waters). Solvent A was 50 mM ammonium formate buffer, pH 4.4, and solvent B was 100% ACN. The gradient used with 130 Å (2.1 × 150 mm, 1.7 μm) column was 0 to 35 min, 12 to 47% solvent A; 35 to 35.5 min, 47 to 70% solvent A; 35.5 to 36.0 min, 70% solvent A; 36 to 36.5 min, 70 to 12% solvent A; 36.5 to 40 min, 12% solvent A. The flow rate was 0.4 ml/min. The injection volume was 3 μl and the sample was prepared in 100% (v/v) ACN. Samples were kept at 5 °C prior to injection, and the separation temperature was 40 °C. The fluorescence detection wavelengths for procainamide were λ_ex_ = 308 nm and λ_em_ = 359 nm, with a data collection rate of 20 Hz.

Conditions for inline mass detection using the ACQUITY quadrupole QDa (Waters) were as follows: Electrospray ionization in positive mode; capillary voltage, 1.5 kV; cone voltage, 15 V; sampling frequency, 5 Hz; probe temperature 400 °C. The QDa analysis was performed using full scan mode, and the mass range was set at *m*/*z* 50 to 800. Single-ion recording mode was used as well to monitor individual glycans. Waters Empower 3 chromatography workstation software was used for data processing, including traditional integration algorithm, no smoothing of the spectra, and manual peak picking.

### Family analysis of GlcNAc-PDase

Protein sequences of EEP superfamily members were obtained from the following sources: [1] UniProt entries for mouse PGAP2IP (Q91YL7), *Saccharomyces cerevisiae* Cwh43p (P25618), and *Schizosaccharomyces pombe* Cwh43p (Q9HDZ2); [2] BLASTP hits from bacteria, archaea, and eukarya using GlcNAc-PDase and Q91YL7 as queries to the NCBI nr database were separately collected until the sets of hits to the two queries overlapped, after which duplicates were removed; [3] BLASTP hits to WP_159773962 (jacalin-related lectin EEP domain) from bacteria, archaea, and eukarya (36); [4] representative proteins from each domain in NCBI’s Conserved Domain Database that is a subset of cd08372 (EEP domain).

Protein sequences from the sources above were combined and redundancy was reduced using CD-Hit (37) to replace sets of sequences sharing >70% identity with a single representative. Sequences that appeared to be incomplete, either based on annotation or on multiple sequence alignment, were removed. The final set comprised 900 sequences.

Sequences were clustered in two dimensions using CLANS (https://toolkit.tuebingen.mpg.de/tools/clans) (38) run under the MPI Bioinformatics Toolkit (39). BLAST connections with *p*-values <1e-04 are shown as edges, and the clustering was run to convergence (typically 10,000–30,000 iterations). Sequences from the GlcNAc-PDase cluster were aligned with MUSCLE v. 5.1 (40) (Fig. S4) within the Geneious software package, regions outside the EEP domain (which in the case of GlcNAc-PDase comprised N-terminal residues 1–53) were removed, and the 186 GlcNAc-PDase EEP domain sequences were reclustered using the same procedure.

### Specificity of GlcNAc-PDase and related proteins

Three closely related gene sequences to GlcNAc-PDase (NLD61619, MBE6669927, and HCG67795) were identified from the Clostridia group of the GlcNAc-PDase family analysis (see above). Signal peptide sequences predicted using SignalP 5.0 (41) were removed from each gene sequence. Gene constructs were synthesized, *E. coli* optimized, and cloned into pUC57 (GenScript). Each sequence was produced *in vitro* using the PURExpress *In vitro* Protein Synthesis Kit as described above (Fig. S5*A*). Activity of each protein was assayed on 4MU-β-GlcNAc-6-PC as described for GlcNAc-PDase (Fig. S5*B*).

The specificity of GlcNAc-PDase and related proteins from the Clostridia group was assessed by incubating *in vitro* produced enzyme with the following custom synthesized monosaccharides: GlcNAc-6-PC, Glc-6-PC, Gal-6-PC, Man-6-PC, GlcNAc-6-PE, Glc-6-PE, Man-6-PE, and Man-2-PE. Reactions were performed in triplicate using 1 μl of *in vitro* produced protein, 10 nmol of each monosaccharide, 50 mM Tris–HCl pH 8, and 5 mM MgCl_2_ in a final volume of 10 μl. After 1 h incubation at 37 °C, 5 μl of each reaction was mixed with 20 μl of 100% ACN and dried using a Vacufuge Plus for 30 min. Dried reactions were procainamide-labeled and resolved on the UPLC as described above.

The specificity of GlcNAc-PDase was also examined by assaying purified *in vivo* produced GlcNAc-PDase-8His with the monosaccharides GlcNAc-6-PC, Glc-6-PC, GlcNAc-6-PE, and Glc-6-PE. Reactions were performed using 4 U of GlcNAc-PDase-8His, 10 nmol of monosaccharide, 50 mM Tris–HCl pH 8, and 5 mM MgCl_2_ in a final volume of 10 μl. After overnight incubation at 37 °C, 5 μl of each reaction was mixed with 20 μl of 100% ACN. The reactions were dried and procainamide-labeled as described above. Reactions were diluted 10-fold and resolved on the UPLC as described above.

### Structural analysis of GlcNAc-PDase and related proteins

Structural predictions of GlcNAc-PDase and the three homologs described above were performed using the Colabfold platform (33) with AlphaFold2 (32) and substituted homology detection and sequence alignment pairing with MMSeq2 (https://github.com/soedinglab/MMseqs2) (42) and HHsearch (https://github.com/soedinglab/hh-suite) (43). Multiple sequence alignment coverage and predicted Local Distance Difference Test plots are provided in Fig. S17. PyMOL by Schrödinger was used for visualizations and interpretation (44). The termini, that include the secretion signal sequence, were predicted with low confidence. Superposition of the GlcNAc-PDase structured region (residues 50–262) with the *Bc*-SMase-Co^2+^ complex (PDB ID 2DDS) was performed for Cα atoms using the *align* command with default parameters for an RMSD of 3.9 Å (157 atoms).

### Extraction and release of *B. malayi* N-linked and GSL glycans

Purified glycans from the parasitic filarial nematode *B*. *malayi* were generated in a previous study (9). In summary, parasite glycoproteins and glycolipids from approximately 600 adult female worms were isolated by tissue homogenization in MQ (MilliQ water), methanol (MeOH) and chloroform in a final 4:7:13 ratio of MQ:MeOH:chloroform. Samples were sonicated and centrifuged at 4000 rpm for 5 min and the upper phase was removed and replaced by 50% MeOH. This step was repeated twice and (glyco)lipids from the upper phase of the extraction were subsequently purified using octadecylsilane (C18) cartridges (BAKERBOND spe, JT Baker, Avantor) as described elsewhere (45). Glycolipids eluted from cartridges were dried under nitrogen (N_2_) flow and reconstituted in 500 μl 50 mM sodium acetate pH 5 containing 0.1% natrium taurodeoxycholate. To release GSL-derived glycans, lipid extracts were treated with a total of 32 mU of recombinant EGCase I over 48 h at 37 °C. An aliquot (16 mU) of the enzyme was first added, while another 16 mU of EGCase I was added after 24 h of incubation. Simultaneously, (glyco-)proteins in the lower phase from the protein/lipid separation procedure described above were pelleted using excess volumes of MeOH. Protein pellets were dried under N_2_ flow and then homogenized in PBS with 1.3% SDS and 0.1% β-mercaptoethanol. Denaturation was performed for 10 min at 95 °C, samples were cooled to room temperature, and Nonidet P-40 (1.3% final concentration) was added to each sample. N-linked glycans were released using 3500 U of PNGase F and incubation at 37 °C for 24 h.

Following their enzymatic release, N-linked and GSL glycans were cleaned-up sequentially on C18 and carbon cartridges (Supelclean ENVI-Carb SPE, MilliporeSigma) according to the protocol previously described (45). Eluted glycans were dried using a Vacufuge plus. The glycans were labeled with 2-aminobenzoic acid (2-AA) by reductive amination with sodium cyanoborohydride as described (46). Labeling was performed for 2 h at 65 °C. To remove labeling reagent excess, ACN was added to a final concentration of 75% and the sample loaded onto Bio-Gel P10 Gel resin (Bio-Rad) previously conditioned with 80% ACN. Glycans were eluted with MQ and dried using a Vacufuge plus.

### Glycan purification using UPLC fractionation

Released and 2-AA–labeled glycans were next purified using a bidimensional UPLC protocol as part of the glycan microarray construction procedure detailed previously (9). Briefly, glycans were separated using the Dionex UltiMate 3000 system (Thermo Fisher Scientific Inc), first by normal phase UPLC using hydrophilic interaction chromatography and then by reverse-phase UPLC on a C18 columns. Fractions were collected, dried down in a Vacufuge plus, and redissolved in 50 μl of MQ. Glycan content was analyzed by MALDI-TOF-MS as described below. *B. malayi* glycans have been structurally characterized using a combination of orthogonal approaches including glycan sequencing procedures and tandem mass spectrometry that have been extensively described previously (9). We selected four fractions shown previously to contain zwitterionic glycans representative of the different contexts of PC-substitution identified in *B. malayi* glycans (9).

### Digestion of zwitterionic *B. malayi* glycans with the GlcNAc-PDase

The four fractions containing PC-substituted glycans were subjected to digestion with GlcNAc-PDase alone or in combination with another exoglycosidase, either β-*N*-acetylhexosaminidase_f_ or the α1-2,3,6 mannosidase. Digestions were performed by mixing 1 to 2 μl (out of 50 μl of the glycan fractions) with 1 μl of GlycoBuffer 1 (5 mM CaCl_2_, 50 mM sodium acetate, pH 5.5 at 25 °C) and with 0.5 μl of the PURExpress reaction mixture containing the *in vitro* produced GlcNAc-PDase. For selected fractions, 2 μl of either β-*N*-acetylhexosaminidase_f_ or α1-2,3,6 mannosidase was additionally used. Reaction volumes were then adjusted to 10 μl total with water and digestions were performed overnight at 37 °C. Negative controls including all the listed reagents minus the glycosidase(s) were run.

Enzyme removal was subsequently performed for all digestion reactions and controls using C18 Millipore Zip-Tips (MilliporeSigma) as detailed previously (47). As described, glycans were directly eluted onto the MALDI plate in 50% ACN, 0.1% trifluoroacetic acid mixed with 10 mg/ml DHB at the end of the clean-up procedure, and were subsequently analyzed using MALDI-TOF-MS.

### MALDI-TOF-MS analysis

MALDI-TOF-MS analysis was performed using an UltrafleXtreme mass spectrometer (Bruker Daltonics) equipped with 1 kHz Smartbeam II laser technology and controlled by the software FlexControl 3.4 Build 119 (Bruker Daltonics). Samples eluted from Zip-Tips were spotted on a 384-well steel polished target plate in DHB matrix as mentioned above. All spectra were obtained in the negative-ion reflectron mode using Bruker peptide calibration standard mix for external calibration. Spectra were obtained over a mass window of *m*/*z* 700 to 3500 with ion suppression below *m*/*z* 700 for a minimum of 20,000 shots (2000 Hz) obtained by manual selection of “sweet spots.” The software flexAnalysis (version 3.4, Build 50, Bruker Daltonics) was used for data processing including smoothing of the spectra (Savitzky Golay algorithm, peak width: *m*/*z* 0.06, 1 cycle), baseline subtraction (Tophat algorithm), and manual peak picking. Peaks with a signal-to-noise ratio below 5 were excluded as well as known nonglycan peaks such as glucose polymers and contaminants introduced by enzyme treatment. Deprotonated masses of the selected peaks were matched to corresponding glycan structures that were previously elucidated (9).

## Data availability

The sequencing data supporting the conclusions of this article are available in the GenBank/EBI Data Bank repository, (OQ439824-OQ439830 in https://www.ncbi.nlm.nih.gov/genbank/). Annotated MALDI-TOF-MS spectra supporting our findings have been made available in the manuscript (main text and supplementary data). In addition, all raw MALDI-TOF-MS data have been deposited in the public repository Glycopost (https://glycopost.glycosmos.org/, project ID: GPST000347).

## Supporting information

This article contains supporting information.

## Conflict of interest

The authors declare that they have no conflicts of interest with the contents of this article.
