# Supplementary material for: A novel family of sugar-specific phosphodiesterases that remove zwitterionic modifications of GlcNAc
Source: J Biol Chem. 2023 Nov 7;299(12):105437. doi: 10.1016/j.jbc.2023.105437 (PMC10704324; doi:10.1016/j.jbc.2023.105437)
Supplement: Supporting Figures S1–S17 and Tables S1 and S2 [file mmc1.docx]

**Supporting Information**

**A novel family of sugar-specific phosphodiesterases that remove zwitterionic modifications of GlcNAc**

Samantha L. Fossa^1^, Brian P. Anton^1^, Daniel W. Kneller^1^, Laudine M.C. Petralia^1,2^, Mehul B. Ganatra^1^, Madison L. Boisvert^1^, Saulius Vainauskas^1^, S. Hong Chan^1^, Cornelis H. Hokke^2^, Jeremy M. Foster^1^ and Christopher H. Taron^1†^

^1^Research Department, New England Biolabs, 240 County Road, Ipswich, MA 01938, USA

^2^Department of Parasitology, Leiden University – Center of Infectious Diseases, Leiden University Medical Center, Leiden, The Netherlands

^†^Corresponding Author: Christopher Taron, 240 County Road, Ipswich, MA 01938, USA, Phone: (978) 927-5054, Fax: (978) 921-1350, E-mail: taron@neb.com

**Figure S1. Functional metagenomic screening.**

A human gut microbiome metagenomic fosmid library was screened with the substrate 4MU-β-GlcNAc-6-PC plus an exogenous hexosaminidase (β-*N*-acetylhexosaminidase_f_). Hits from the primary screen were re-screened to determine if the observed activity was reproducible. Shown are fluorescence values at the 24 h timepoint. Metagenomic clones (orange circles) were screened for activity. Replicates of the pCC1 empty fosmid vector (grey circles) were screened as a negative control for background fluorescence. Hits from the re-screen were defined as clones yielding an assay signal at least 10 standard deviations (black line) above the mean background fluorescence.

**Figure S2. Activity of GlcNAc-PDase on 4MU-labeled GlcNAc-6-PC substrate analyzed by UPLC-FLR-MS.**

A) UPLC-FLR chromatograms showing separation of the 4MU-β-GlcNAc-6-PC and 4MU-β-GlcNAc before and after incubation with GlcNAc-PDase. Peaks detected due to the intrinsic fluorescence of the PURExpress^®^ components are denoted with an asterisk (*)

B) Analysis of the reaction products from panel A (middle and bottom chromatograms) by UPLC with inline MS.


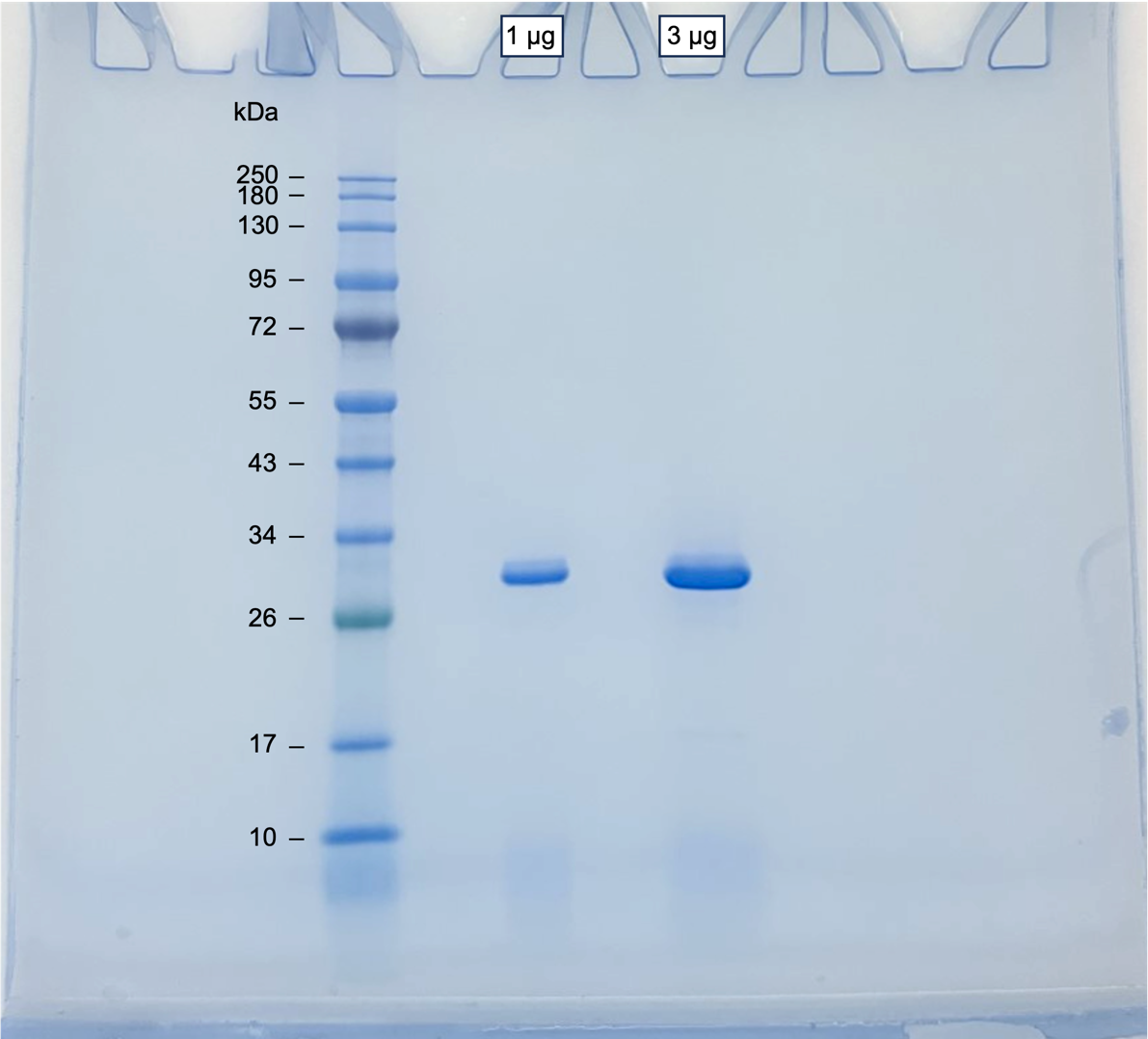


**Figure S3. GlcNAc-PDase-8His purification.**

GlcNAc-PDase-8His was expressed from the P*tac* promoter in *E. coli* and purified using Ni-NTA affinity chromatography. The theoretical molecular weight of GlcNAc-PDase is 29.1 kDa. Purified protein (1µg and 3µg) was loaded on the SDS-PAGE and stained using SimplyBlue™ SafeStain.

**
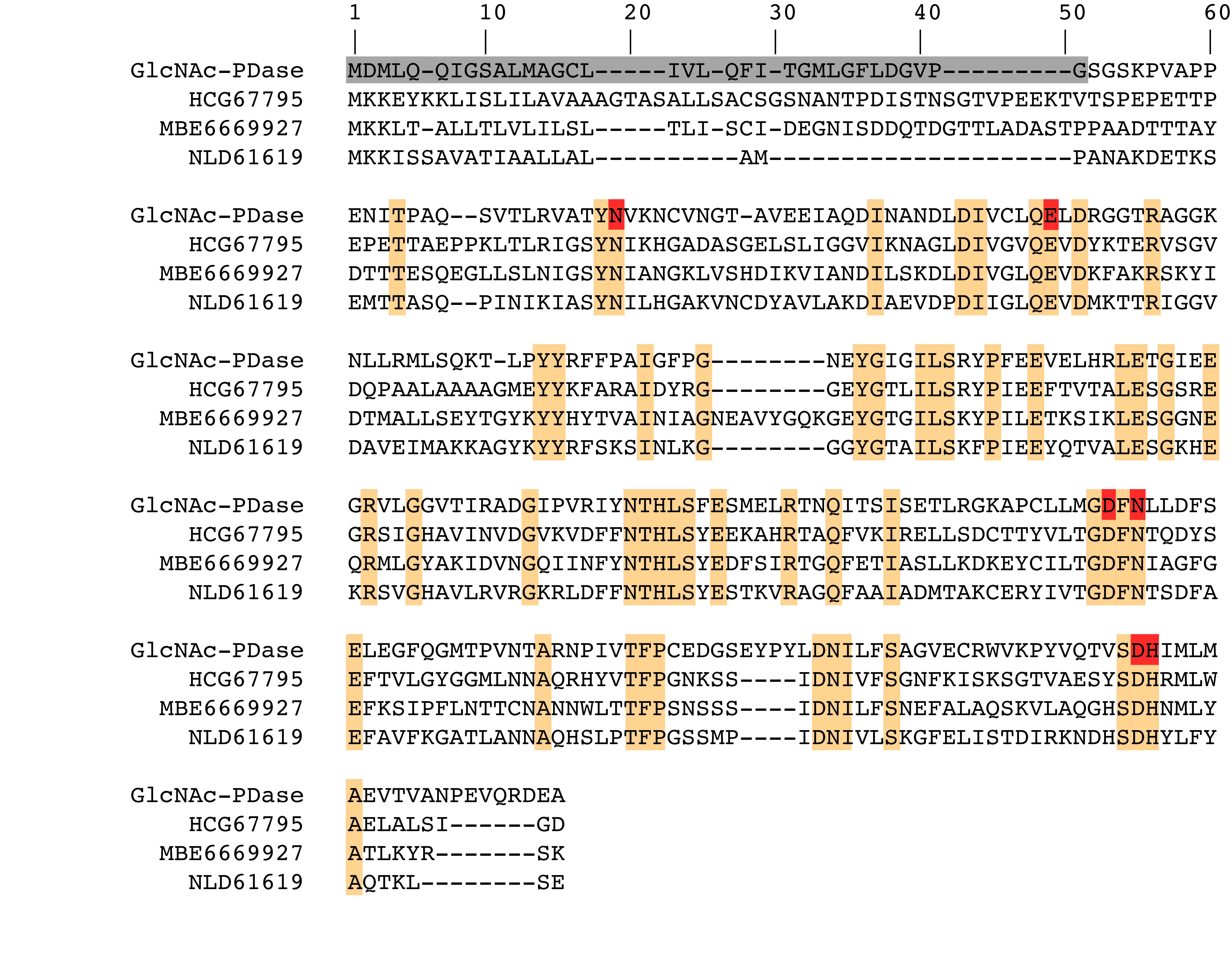
**

**Figure S4. GlcNAc-PDase and related “Clostridia group” proteins alignment.**

The deduced peptide sequence of GlcNAc-PDase was aligned with three proteins (GenBank numbers: HCG67795, MBE6669927, and NLD61619) from the Clostridia group using the program MUSCLE ([https://www.ebi.ac.uk/Tools/msa/muscle/](https://nam10.safelinks.protection.outlook.com/?url=https%3A%2F%2Fwww.ebi.ac.uk%2FTools%2Fmsa%2Fmuscle%2F&data=05%7C01%7Csfossa%40neb.com%7Ce6059baeff784958808808db515a8913%7C77cefbc6b3d64d6a9f740664881c384b%7C0%7C0%7C638193221540365787%7CUnknown%7CTWFpbGZsb3d8eyJWIjoiMC4wLjAwMDAiLCJQIjoiV2luMzIiLCJBTiI6Ik1haWwiLCJXVCI6Mn0%3D%7C3000%7C%7C%7C&sdata=Uv4rWd6y%2FKdXZ9LRNQnGUuuZ4NuiWhj3iQSMePUnj2o%3D&reserved=0)). Identical residues are shaded orange and gaps introduced into the alignment by the algorithm are denoted with a hyphen. The predicted signal peptide for GlcNAc-PDase (aa 1-34) is shaded gray and GlcNAc-PDase residues shaded in red were mutated to alanine to probe involvement in catalytic activity (Figure 5D).

A

B

**Figure S5. Expression and activity of “Clostridia group” related proteins.**

A) Three related proteins from the Clostridia group (NLD61619, MBE6669927, and HCG67795) were expressed by *in vitro* transcription and translation (IVTT) using the PURExpress^®^ system (red circles) and compared to GlcNAc-PDase. An empty vector (pCC1) negative control and a PURExpress^®^ DHFR (dihydrofolate reductase) positive control plasmid were also processed.

B) IVTT-produced material was assayed for fluorescence activity on the screening substrate 4MU-β-GlcNAc-6-PC with or without supplemented β-hexosaminidase.

**Figure S6. GlcNAc-PDase and “Clostridia group” protein activity on GlcNAc-6-PC with UPLC-FLR analysis.**

IVTT-produced (A) NLD61619 (related protein 1), (B) MBE6669927 (related protein 2), (C) HCG67795 (related protein 3), (D) GlcNAc-PDase and (E) a negative control were incubated with the monosaccharide *N*-acetyl-D-glucosamine-6-phosphorylcholine (GlcNAc6PC) in triplicate. Reactions were procainamide labeled and separated using UPLC-FLR.

**Figure S7. GlcNAc-PDase and “Clostridia group” protein activity on Glc-6-PC with UPLC-FLR analysis.**

IVTT-produced (A) NLD61619 (related protein 1), (B) MBE6669927 (related protein 2), (C) HCG67795 (related protein 3), (D) GlcNAc-PDase and (E) a negative control were incubated with the monosaccharide 6-O-phosphorylcholine-D-glucopyranose (Glc-6-PC) in triplicate. Reactions were procainamide labeled and separated using UPLC-FLR.

**Figure S8. GlcNAc-PDase and “Clostridia group” protein activity on Gal-6-PC with UPLC-FLR analysis.**

IVTT-produced (A) NLD61619 (related protein 1), (B) MBE6669927 (related protein 2), (C) HCG67795 (related protein 3), (D) GlcNAc-PDase and (E) a negative control were incubated with the monosaccharide 6-O-phosphorylcholine-D-galactopyranose (Gal-6-PC) in triplicate. Reactions were procainamide labeled and separated using UPLC-FLR.

**Figure S9. GlcNAc-PDase and “Clostridia group” protein activity on Man-6-PC with UPLC-FLR analysis.**

IVTT-produced (A) NLD61619 (related protein 1), (B) MBE6669927 (related protein 2), (C) HCG67795 (related protein 3), (D) GlcNAc-PDase and (E) a negative control were incubated with the monosaccharide 6-O-phosphorylcholine-D-mannopyranose (Man-6-PC) in triplicate. Reactions were procainamide labeled and separated using UPLC-FLR.

**Figure S10. GlcNAc-PDase and “Clostridia group” protein activity on GlcNAc-6-PE with UPLC-FLR analysis.**

IVTT-produced (A) NLD61619 (related protein 1), (B) MBE6669927 (related protein 2), (C) HCG67795 (related protein 3), (D) GlcNAc-PDase and (E) a negative control were incubated with the monosaccharide *N*-acetyl-D-glucosamine-6-phosphoethanolamine (GlcNAc-6-PE) in triplicate. Reactions were procainamide labeled and separated using UPLC-FLR.

**Figure S11. GlcNAc-PDase and “Clostridia group” protein activity on Glc-6-PE with UPLC-FLR analysis.**

IVTT-produced (A) NLD61619 (related protein 1), (B) MBE6669927 (related protein 2), (C) HCG67795 (related protein 3), (D) GlcNAc-PDase and (E) a negative control were incubated with the monosaccharide 6-O-phosphoethanolamine-D-glucopyranose (Glc-6-PE) in triplicate. Reactions were procainamide labeled and separated using UPLC-FLR.

**Figure S12. GlcNAc-PDase and “Clostridia group” protein activity on Man-6-PE with UPLC-FLR analysis.**

IVTT-produced (A) NLD61619 (related protein 1), (B) MBE6669927 (related protein 2), (C) HCG67795 (related protein 3), (D) GlcNAc-PDase and (E) a negative control were incubated with the monosaccharide 6-O-phosphoethanolamine-D-mannopyranose (Man-6-PE) in triplicate. Reactions were procainamide labeled and separated using UPLC-FLR.

**Figure S13. GlcNAc-PDase and “Clostridia group” protein activity on Man-2-PE with UPLC-FLR analysis.**

IVTT-produced (A) NLD61619 (related protein 1), (B) MBE6669927 (related protein 2), (C) HCG67795 (related protein 3), (D) GlcNAc-PDase and (E) a negative control were incubated with the monosaccharide 2-O-phosphoethanolamine-D-mannopyranose (Man-2-PE) in triplicate. Reactions were procainamide labeled and separated using UPLC-FLR.

**Figure S14. Structural conservation analysis of GlcNAc-PDase with related proteins.**

A) Structural predictions of GlcNAc-PDase homologs NLD61619, MBE6669927, and HCG67795 in cartoon representation colored per residue by predicted Local Distance Difference Test (pLDDT) score. The structured region of the top 5 ranks are shown superimposed to rank 1 by global alignment of C-alpha atoms.

B) Superposition of GlcNAc-PDase and related proteins demonstrate a consistent secondary structure folding pattern with apparent conserved metal coordinating residues at locations consistent with experimentally determined EEP enzyme *Bc*-SMase.

**Figure S15. *B. malayi* GSL glycans digestion with β-*N*-acetylhexosominidase_f_ and GlcNAc-PDase.**

Endoglucoceramidase (EGCase I; New England Biolabs)-released, AA-labeled and UPLC-purified *B. malayi* GSL glycans were subjected to digestion with GlcNAc-PDase (A.ii), β-*N*-acetylhexosominidase_f_ (B.i) or a combination of both enzymes (B.ii). Resulting digestion product is highlighted using a red dashed arrow. MALDI-TOF-MS spectra monoisotopic masses are indicated for ions with signal-to-noise ratio above 5. Non-glycan peaks are shown using grey stars and glycan structures are represented using the CFG nomenclature. See Symbol key inset. MALDI-TOF-MS raw data can be found in Table S2.B in the separate Excel file named “Table S2”.

**Figure S16. *B. malayi* N-glycans digestion with α1-2,3,6 mannosidase and GlcNAc-PDase.**

PNGase F-released, AA-labeled and UPLC-purified *B. malayi* N-glycans were subjected to digestion with GlcNAc-PDase (A.ii), α1-2,3,6 mannosidase (B.i) or a combination of both enzymes (B.ii). MALDI-TOF-MS spectra monoisotopic masses are indicated for ions with signal-to-noise ratio above 5. Glycan structures are represented using the CFG nomenclature. See Symbol key inset. Non-glycan peaks are signaled using grey stars. MALDI-TOF-MS raw data can be found in Table S2.C in the separate Excel file named “Table S2”.

**
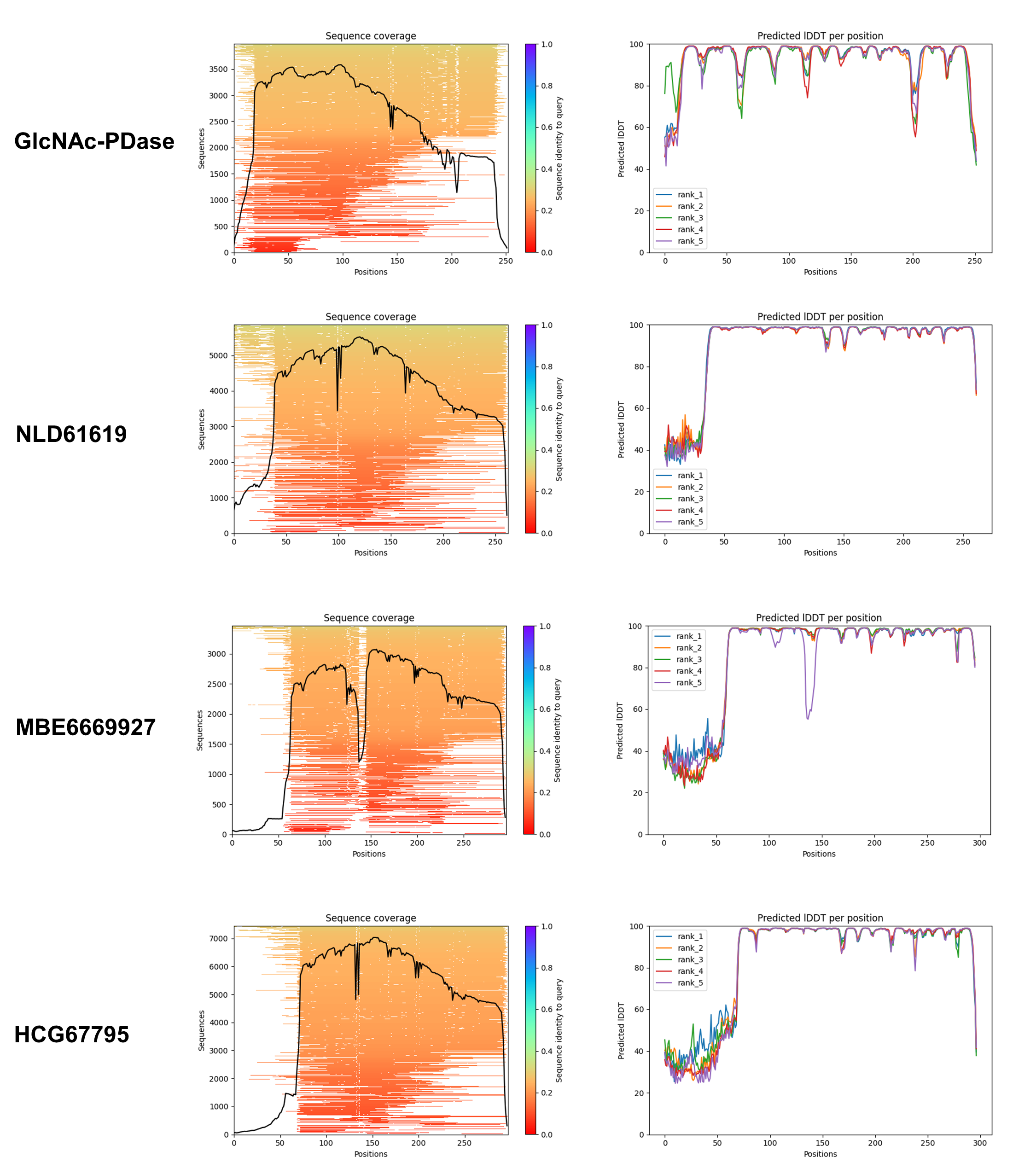
**

**Figure S17. MSA and pLDDT plots from ColabFold predictions.**

Multiple sequence alignment (MSA) coverage and predicted Local Distance Difference Test (pLDDT) plots from ColabFold structural predictions for GlcNAc-PDase, NLD61619, MBE6669927, and HCG67795.

**Table S1. Primers used in this study.**

| **Primer** | **Sequence** | **Use** |
| --- | --- | --- |
| PURExpress_F1-ORF15_Fwd | GCGAATTAATACGACTCACTATAGGGCTTAAGTATAAGGAGGAAAAAATATGGAAGTAATACTCATTCGACACACT | *In vitro* expression of ORF1 from fosmid F1 |
| PURExpress_F1-ORF15_Rev | AAACCCCTCCGTTTAGAGAGGGGTTATGCTAGTTATTCCTTGTCGAGATTGATGCGGAC |  |
| PURExpress_F2-ORF23_Fwd | GCGAATTAATACGACTCACTATAGGGCTTAAGTATAAGGAGGAAAAAATATGAAAGAGAACAAACCCTGCCTTTTG | *In vitro* expression of ORF23 from fosmid F2 |
| PURExpress_F2-ORF23_Rev | AAACCCCTCCGTTTAGAGAGGGGTTATGCTAGTTAATTTCCACATGATCTGTTTTCTAT |  |
| PURExpress_F3-ORF6_Fwd | GCGAATTAATACGACTCACTATAGGGCTTAAGTATAAGGAGGAAAAAATATGGCATTTCTCGAAAGGAGCCCGGCT | *In vitro* expression of ORF6 from fosmid F3 |
| PURExpress_F3-ORF6_Rev | AAACCCCTCCGTTTAGAGAGG GGTTATGCTAGTTAAGCCTCCATCAGCACTTTCCCGTC |  |
| PURExpress_F4-ORF27_Fwd | GCGAATTAATACGACTCACTATAGGGCTTAAGTATAAGGAGGAAAAAATATGGATATGTTGCAGCAAATTGGGAGC | *In vitro* expression of ORF27 from fosmid F4 |
| PURExpress_F4-ORF27_Rev | AAACCCCTCCGTTTAGAGAGGGGTTATGCTAGTTAAGCTTCATCCCGCTGCACCTCCGG |  |
| PURExpress_F5-ORF14_Fwd | GCGAATTAATACGACTCACTATAGGGCTTAAGTATAAGGAGGAAAAAATATGAGCAAGGAATCCGTAACCGTCGCC | *In vitro* expression of ORF14 from fosmid F5 |
| PURExpress_F5-ORF14_Rev | AAACCCCTCCGTTTAGAGAGGGGTTATGCTAGTTATTGAGCCAGCAACCGGCGACCATA |  |
| PURExpress_F5-ORF23_Fwd | GCGAATTAATACGACTCACTATAGGGCTTAAGTATAAGGAGGAAAAAATATGGCAGTGGTGTTGGCCGGCGTGGCA | *In vitro* expression of ORF23 from fosmid F5 |
| PURExpress_F5-ORF23_Rev | AAACCCCTCCGTTTAGAGAGGGGTTATGCTAGTTAGGCCAGTCCCATCTTGATTTTCAA | *In vitro* expression of ORF23 from fosmid F5 |
| PURExpress_F6-ORF3_Fwd | GCGAATTAATACGACTCACTATAGGGCTTAAGTATAAGGAGGAAAAAATATGAACGATAATAAAAACAGTATGAAA | *In vitro* expression of ORF3 from fosmid F6 |
| PURExpress_F6-ORF3_Rev | AAACCCCTCCGTTTAGAGAGG GGTTATGCTAGTTATAGCGCTCCCCTACGCAATATGTC |  |

| PURExpress_F7-ORF3_Fwd | GCGAATTAATACGACTCACTATAGGGCTTAAGTATAAGGAGGAAAAAATATGTCTGCAGCAAAAAGCAGTGACCTG | *In vitro* expression of ORF3 from fosmid F7 |
| --- | --- | --- |
| PURExpress_F7-ORF3_Rev | AAACCCCTCCGTTTAGAGAGGGGTTATGCTAGTTATAAATATTTCACGCCCCAGACACT |  |
| PURExpress_NLD61619_Fwd | GCGAATTAATACGACTCACTATAGGGCTTAAGTATAAGGAGGAAAAAATATGAAAGATGAGACAAAGTCAGAAATG | *In vitro* expression of NLD61619 |
| PURExpress_NLD61619_Rev | AAACCCCTCCGTTTAGAGAGGGGTTATGCTAGTTATTCAGACAATTTGGTTTGCGCATA |  |
| PURExpress_MBE6669927_Fwd | GCGAATTAATACGACTCACTATAGGGCTTAAGTATAAGGAGGAAAAAATATGATTGATGAAGGAAATATATCAGAT | *In vitro* expression of MBE6669927 |
| PURExpress_MBE6669927_Rev | AAACCCCTCCGTTTAGAGAGGGGTTATGCTAGTTACTTAGAGCGGTATTTCAGGGTCGC |  |
| PURExpress_HCG67795_Fwd | GCGAATTAATACGACTCACTATAGGGCTTAAGTATAAGGAGGAAAAAATATGTTACTATCAGCTTGTTCTGGAAGT | *In vitro* expression of HCG67795 |
| PURExpress_HCG67795_Rev | AAACCCCTCCGTTTAGAGAGGGGTTATGCTAGTTAGTCGCCGATGGACAGCGCCAACTC |  |
| PURExpress_F4-ORF27mutant_N60A_E89A_N206A_D266A_H267A_Fwd | GCGAATTAATACGACTCACTATAGGGCTTAAGTATAAGGAGGAAAAAATATGGATATGCTACAACAGATAGGATCA | *In vitro* expression of ORF27 mutants from fosmid F4. Forward primer used to generate individual mutants for N60A, E89A, N206A, D266A, and H267A. |
| PURExpress_F4-ORF27mutant_N60A_Rev | AAACCCCTCCGTTTAGAGAGGGGTTATGCT AGTTACGCTTCATCACGCTGCACCTCCGG | *In vitro* expression of ORF27 mutant N60A from fosmid F4 |
| PURExpress_F4-ORF27mutant_E89A-Rev | AAACCCCTCCGTTTAGAGAGGGGTTATGCT AGTTATGCTTCATCACGCTGAACTTCCGG | *In vitro* expression of ORF27 mutant E89A from fosmid F4 |
| PURExpress_F4-ORF27mutant_D204A_Fwd | GCGAATTAATACGACTCACTATAGGGCTTAAGTATAAGGAGGAAAAAATATGGATATGCTACAACAGATAGGAAGT | *In vitro* expression of ORF27 mutant D204A from fosmid F4 |
| PURExpress_F4-ORF27mutant_D204A_Rev | AAACCCCTCCGTTTAGAGAGGGGTTATGCTAGTTATGCCTCATCACGTTGGACTTCCGG |  |
| PURExpress_F4-ORF27mutant_N206A_Rev | AAACCCCTCCGTTTAGAGAGGGGTTATGCTAGTTAAGCCTCATCACGCTGCACTTCCGG | *In vitro* expression of ORF27 mutant N206A from fosmid F4 |
| PURExpress_F4-ORF27mutant_D266A_Rev | AAACCCCTCCGTTTAGAGAGGGGTTATGCTAGTTATGCTTCATCACGCTGAACTTCCGG | *In vitro* expression of ORF27 mutant D266A from fosmid F4 |
| PURExpress-F4-ORF27mutant-H267A-Rev | AAACCCCTCCGTTTAGAGAGGGGTTATGCTAGTTACGCCTCATCACGCTGCACCTCCGG | *In vitro* expression of ORF27 mutant H267A from fosmid F4 |
| pJS119k_ORF27_Fwd | GAATTCAGCTTGGCTGTTTTG | Linearization of pJS119k vector for HiFi cloning of ORF27 |
| pJS119k_ORF27_Rev | ATGTTAACCTCCTAAGCTTAATTC |  |
| F4-ORF27_Fwd | TTAAGCTTAGGAGGTTAACATATGGATATGTTGCAGCAAATTGGGAG | Amplifying ORF27 (GlcNAc-PDase) from fosmid F4 with C-terminal 8His for subcloning into pJS119k to generate pJS119k-GlcNAc-PDase-8His |
| F4-ORF27-8His(Cterm)_Rev | CAAAACAGCCAAGCTGAATTCTCAGTGGTGGTGGTGGTGGTGATGATGAGCTTCATCCCGCTGCACCTC |  |
| pJS119K-GlcNAc-PDase-8His_SDM_Fwd | AGCGGCTCAAAGCC | Removal of signal peptide sequence from pJS119k-GlcNAc-PDase-8His by site-directed mutagenesis |
| pJS119K-GlcNAc-PDase-8His_SDM_Rev | CATATGTTAACCTCCTAAGCTTAATTC |  |

**Table S2. MALDI-TOF-MS raw data.**

This table can be found in the separate Excel file named “Table S2”. This file contains raw data for Supporting Information Figures S15 and S16, and Figure 6 of the main text.
